# Effect of RNA integrity on uniquely mapped reads in RNA-Seq

**DOI:** 10.1186/1756-0500-7-753

**Published:** 2014-10-23

**Authors:** Emily A Chen, Tade Souaiaia, Jennifer S Herstein, Oleg V Evgrafov, Valeria N Spitsyna, Danea F Rebolini, James A Knowles

**Affiliations:** Department of Genetic, Molecular, and Cellular Biology, Keck School of Medicine, Zilkha Neurogenetic Institute, University of Southern California, 1501 San Pablo Street, ZNI 401, MC 2821, Los Angeles, CA 90089-2821 USA; Department of Psychiatry and the Behavioral Sciences, Keck School of Medicine, Zilkha Neurogenetic Institute, University of Southern California, 1501 San Pablo Street, ZNI 401, MC 2821, Los Angeles, CA 90089-2821 USA; Illumina, Inc, 5200 Illumina Way, San Diego, CA 92122 USA

**Keywords:** Gene expression, RNA-Sequencing, Low-quality RNA, Library construction protocol, Degradation

## Abstract

**Background:**

We examined the performance of three RNA-Sequencing library preparation protocols as a function of RNA integrity, comparing gene expressions between heat-degraded samples to their high-quality counterparts. This work is invaluable given the difficulty of obtaining high-quality RNA from tissues, particularly those from individuals with disease phenotypes.

**Results:**

With the integrity of total RNA being a critical parameter for RNA-Sequencing analysis, degraded RNA can heavily influence the results of gene expression profiles. We discovered that gene expression read results are influenced by RNA quality when a common library construction protocol is used. These results are based on one technical experiment from a pool of 4 neural progenitor cell lines.

**Conclusions:**

The use of alternative protocols can allow samples with a wider range of RNA qualities to be used, facilitating the investigation of disease tissues.

## Discussion

### Background

*Adiconis et al.* [1] examined the performance of five RNA-Seq sample preparation protocols when using RNA of low quality and/or quantity. This work is invaluable given the difficulty of obtaining high-quality RNA from tissues, particularly those from individuals with disease phenotypes. We have used a similar approach of evaluating the performance of RNA-Seq library preparation protocols, as a function of RNA integrity. We compared gene expression, as measured by RNA-Seq, of heat-degraded RNA samples to the expression profiles of the high-quality starting samples.

### Methods and results

Specifically, 20 ug of high-quality total RNA (RIN 9.4; 2100 Bioanalyzer, Agilent Technologies Inc., Santa Clara, CA, USA) was constructed by pooling RNA extracted using a Direct-zol RNA MiniPrep kit (Zymo Research, Irvine, CA, USA) from neural progenitor cell lines made from 4 individuals [2]. This pool was heat-degraded (60 minutes at 60°C, followed by 6, 20 and 30 mins at 90°C) to RINs of 7.4, 5.3, and 4.5 [3]. RNA-Seq libraries were then made using three different protocols. 1) Poly-A RNA was purified from 1 ug of total RNA using oligo-dT beads, fragmented with divalent cations, made into cDNA and then sequencing libraries using the TruSeq RNA Sample Preparation kit v2 (RS-122-2001, Illumina Inc., San Diego, CA, USA). 2) Ribosomal RNA was removed from 1 ug of total RNA using the Ribo-Zero rRNA Removal kit (MRZH116, Epicentre Biotechnologies, Madison, WI, USA), and processed without the poly-A selection as per #1. 3) cDNA was made from 200 ng of total RNA using the Ovation RNA-Seq FFPE System (7150, NuGEN Technologies Inc., San Carlos, CA, USA), sheared to 300 bp using a Covaris S2 (500003, Covaris Inc., Woburn, MA, USA), and followed by library construction using the TruSeq DNA Sample Preparation kit v2 (FC-121-2001).

**Figure 1:**
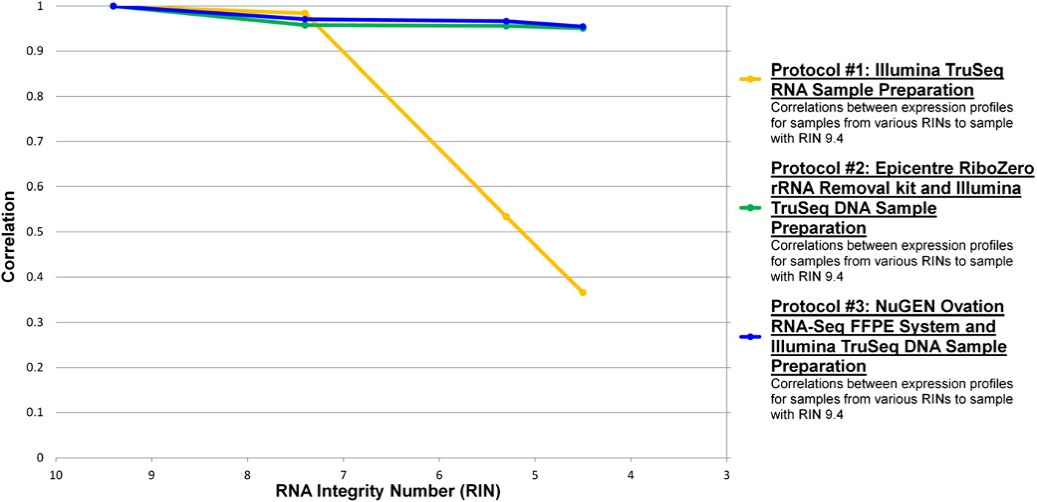
**Effect of RNA integrity on gene expression correlations with untreated RNA using the same sample preparation protocol.**

**Figure 2:**
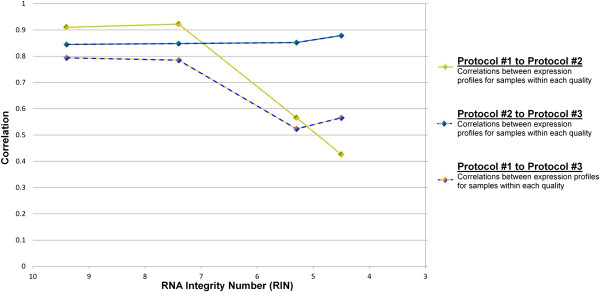
**Effect of RNA integrity on gene expression correlations between different sample preparation protocols.**

Each DNA library was sequenced with 4.5–60 million 100 bp single-end reads on an Illumina HiSeq2000. The reads were uniquely mapped with three or fewer mismatches using PerM [4] to GENCODE v17. For each protocol, we calculated Pearson’s pairwise correlation coefficients (denoted by the letter R) between the degraded and high-quality sample across the HUGO genes which contained at least one read alignment in either sample. R was calculated and depicted in Figure 1 by taking the log of (reads plus an offset of 1). All three protocols performed well at RIN 7.4 (R = 0.958 to 0.984, s.e. = 0.001 to 0.002) (Figure 1, Table 1). However, as RNA quality decreased (RINs 5.3 and 4.5), protocol #1 produced data with lower correlations of gene expression to the intact sample (R = 0.533 and 0.366, s.e. =0.005). In contrast, both protocols #2 and #3 performed relatively well as RNA quality decreased (R = 0.951 to 0.967, s.e. = 0.002), with protocol #3 performing slightly better. For each RIN quality, we calculated R between the reads from each pair of protocols. The reads from the two best methods (Protocol #2 and Protocol #3) maintained high correlations regardless of decreased sample quality (R = 0.845 to 0.879, s.e. = 0.003). For Protocol #1, there was a drop in read correlation to both Protocol #2 and Protocol #3 as RIN decreased (Figure 2).

**Table 1:** **R between degraded sample and intact sample for each protocol**

Protocol	RIN	R to RIN 9.4 sample	Total Reads
NuGEN Ovation RNA-Seq FFPE System + Illumina TruSeq DNA Sample Preparation	9.4	1	37,959,903
	7.4	0.971	51,131,950
	5.3	0.967	32,285,466
	4.5	0.955	58,476,532
Epicentre RiboZero rRNA Removal Kit + Illumina TruSeq DNA Sample Preparation	9.4	1	25,339,734
	7.4	0.958	59,722,682
	5.3	0.956	48,134,260
	4.5	0.951	50,650,245
Illumina TruSeq RNA Sample Preparation	9.4	1	59,509,601
	7.4	0.984	35,262,055
	5.3	0.533	20,939,261
	4.5	0.366	4,531,318

For confirmation of mapper accuracy, we mapped all of the samples using TopHat v1.4.0 [5] to GENCODE v17. The resulting BAM files were run through HTSeq v0.6.1 [6] to obtain uniquely mapped read counts. Essentially the same results were obtained as with PerM (data not shown). Additionally, to rule out any bias from differences in numbers of reads, we downsampled all of the samples to 4.5 million reads, and the results were essentially the same (data not shown).

### Conclusions

It is likely that the poor performance of protocol #1 at lower RINs can be explained by the poly-A selection step. As RNA integrity decreases, less full length poly-A RNA is recovered, leading to a cDNA library that is increasingly 3′ biased. This is supported by analysis of the 5′ to 3′ read distribution of each library. Those from protocols #2 and #3 are essentially unchanged at decreasing RIN, while the distribution for samples from protocol #1 is severely 3′ biased by RIN 4.5 (data not shown).

We recognize that our results are based on a single experiment using an RNA pool from 4 neural progenitor cell lines and are not broadly applicable. Hence, other investigators may want to use this method to determine the effect of RNA integrity on RNA-Seq from their tissue source of interest.

In summary, our data show that the results of RNA-Seq are influenced by RNA quality with a widely-used cDNA/sequencing library construction protocol. However, this problem can be avoided with alternative protocols, allowing samples with a wider range of RNA qualities to be used, facilitating the investigation of disease tissues.

## References

[1] Adiconis X, Borges-Rivera D, Satija R, DeLuca DS, Busby MA, Berlin AM, Sivachenko A, Thompson DA, Wysoker A, Fennell T, Gnirke A, Pochet N, Regev A, Levin JZ (2013). Comparative analysis of RNA sequencing methods for degraded or low-input samples. Nat Methods.

[2] Evgrafov OV, Wrobel BB, Kang X, Simpson G, Malaspina D, Knowles JA (2011). Olfactory neuroepithelium-derived neural progenitor cells as a model system for investigating the molecular mechanisms of neuropsychiatric disorders. Psychiatr Genet.

[3] Opitz L, Salinas-Riester G, Grade M, Jung K, Jo P, Emons G, Ghadimi BM, Beissbarth T, Gaedcke J (2010). Impact of RNA degradation on gene expression profiling. BMC Med Genomics.

[4] Chen Y, Souaiaia T, Chen T (2009). PerM: efficient mapping of short sequencing reads with periodic full sensitive spaced seeds. Bioinformatics.

[5] Trapnell C, Pachter L, Salzberg S (2009). TopHat: discovering splice junctions with RNA-Seq. Bioinformatics.

[6] Anders S, Pyl PT, Huber W (2014). HTSeq - A Python framework to work with high-throughput sequencing data. BioRxiv preprint.

